# Lncap-AI prostate cancer cell line establishment by Flutamide and androgen-free environment to promote cell adherent

**DOI:** 10.1186/s12860-022-00453-2

**Published:** 2022-11-28

**Authors:** Huifeng Wang, Xihua Wei, Die Zhang, Weidong Li, Yanling Hu

**Affiliations:** 1 Department of Human Anatomy, School of Basic Medical Sciences, Guangxi Medical University, 530021 Nanning, Guangxi China; 2 Key Laboratory of Human Development and Disease Research, Education Department of Guangxi Zhuang Autonomous Region, Guangxi Medical University, Guangxi 530021 Nanning, China; 3 Institute of Life Sciences, Guangxi Medical University, 530021 Nanning, Guangxi China; 4 Collaborative Innovation Centre of Regenerative Medicine and Medical BioResource Development and Application Co-constructed by the Province and Ministry, Guangxi Medical University, 530021 Nanning, Guangxi China

**Keywords:** Prostate Cancer, CRPC, Androgen Independent, ADT, Androgen resistant

## Abstract

**Background:**

To establish castration-resistant prostate cancer (CRPC) - Lncap androgen-independent (AI) cell line from Lncap androgen-dependent (AD) cell line, and explore the different molecular biological between these two cell lines.

**Methods:**

The Lncap-AD cell line was cultured and passaged 60 times over 16 months. The morphology of the Lncap-AI cell line was observed. AR levels identification were detected in qRT-PCR and Western Blot assay. CCK-8, EdU assay, wound healing assay and cell adhesion assays were used to observe the ability of proliferation, migration, and adhesion. SEM and TEM were used to observe microculture structure. At last, the PSA secrete ability was evaluated by Elisa assay.

**Results:**

The Lncap-AD cell line was cultured and passaged 60 times over 16 months. The Lncap-AI cell line showed a morphologic change at the end stage of culture, the cells turned slender and cell space turned separated compared to the Lncap-AD cell line. The relative levels of AR-related genes in the Lncap-AI cell line were up-regulation compared to the Lncap-AD cell line both in mRNA and protein levels. The expression of AR and HK2 proteins were influenced and down-regulation by Enzalutamide in the Lncap-AD cell line, but no obvious difference in Lncap-AI cell lines. Lncap-AI cell line showed strong viability of proliferation, migration, and adhesion by CCK-8, EdU assay, wound healing assay, and adhesion assay. The microstructure of Scanning Electron Microscopy (SEM) showed many synapses in the Lncap-AI cell line and PC3 cell line, but not in the Lncap-AD cell line. At last, the PSA secrete ability was evaluated by Elisa assay, and PCa cell lines showed no significant difference.

**Conclusion:**

Simulation of CRPC progression, Lncap-AD cell line turned to Lncap-AI cell line with androgen deprivation therapy.

**Supplementary Information:**

The online version contains supplementary material available at 10.1186/s12860-022-00453-2.

## Background

Prostate cancer (PCa) is the world’s top 10 malignancy disease among men which causes leading to male-related cancer death in most countries [1]. The earlier stage of PCa can be diagnosed by magnetic resonance imaging technology and with the popularization of early prostate-specific antigen (PSA) screening [2]. At this time, the tumor is also confined to the prostate tissue and could achieve a good result using surgery and radiation therapy [3]. Androgen deprivation therapy (ADT) caused good therapy efficiency at an early time. However, during the stage of ADT, the PCa will progress from androgen dependent to androgen independent which is called castration resistant prostate cancer (CRPC). However, patients with castration resistant prostate cancer (CRPC) have a poor prognosis because the efficacy of hormone deprivation therapy is only temporary, and most patients develop hormone non-dependence after 6–18 months of androgen deprivation therapy (ADT) [4]. Once PCa enters a hormone-non-dependent state, cancer cells can resist tumor suppression caused by ADT. There is no effective treatment for these patients to prolong their survival [5].

There are several prostate cancer cell models including androgen-dependent and androgen-independent models, respectively. The PCa cells, such as PC3 and DU145, do not express androgens, and these cell lines can grow in a hormone-deprived climate. Currently, only the Lncap cell line retains PCa androgen-dependent characteristics in humans that secrete PSA, PsMA, and androgens, increasing by sustaining steroid hormones. Lncap is, therefore, the most commonly used prostate cancer cell line. However, it is hard to explore how prostate cancer’s mechanism details progress in a dependent state compared to a non-dependent progression state of androgen. Although some studies provide valuable information on the mechanisms by which androgen is not dependent on PCa in many ways [6–8]. But the molecular biology of PCa transition from androgen-dependent to androgen-independent is not yet well understood. One major research obstacle is the lack of an ideal cell model. A perfect PCa cell model should show androgen-dependent properties that express androgen receptors transformed into androgen-independent in an androgen-deprived environment.

Cell adhesion is the correlation between cells and molecules of extracellular mechanisms. Cell adhesion function could be used in multiple physiological and pathological processes, such as embryonic development and differentiation, maintenance of normal tissue structure, inflammatory response, immune response, clotting and thrombosis, trauma repair, tumor immersion and metastasis, and embryo occurrence. Cell adhesion is also an important character in the hallmark of cancer, which has a strong relationship with metastasis [9–11]. At present, the cell adhesion mechanism has become one of the hot spots in cancer cell invasion and metastasis research. In some PCa studies, cell adhesion could influence metastasis by regulating E-Cadherin [9], GRP78, amygdalin, etc. ,This study aimed to establish an androgen-independent Lncap (LncapAI) cell subsystem from an androgen-dependent Lncap (LncapAD) cell line during a castration androgen environment and explore a more appropriate method to improve the culture protocol and advance the efficiency to establish the Lncap-AI cell line.

## Methods

### Cell culture

Lncap cell line (5 passages, provided by the FuHeng Ltc., Shanghai, China) was digested with 1 mL 0.25% Trypsin Digestion solutions with EDTA (Solarbio, catalog number: T1350) at 37 °C for 50 s. The cells were cultured with androgen deprivation condition in culture medium RPMI Medium 1640(gibco, catalog number: 11835-030) with 10% Certified Foetal Bovine Serum (FBS) Charcoal Stripped (Biological Industries Pricelist, catalog number: 04-201-1 A), 1% GlutaMAX-I (gibco, catalog number: 35050-061) and 1% Sodium Pyruvate (gibco, catalog number: 11360-070). The culture medium was replaced every five days. Incubator until the cells get reach ~ 80% confluence to cell freezing or cell passage.

### Cell treatment and cell morphology

The cells were placed in a 10-cm culture dish and co-cultured with Flutamide (Yuanye, Shanghaiyuanye Bio-Technology™, catalog number: H20A9Z68074), and sustained culture 60–80 passages at 37 °C. Flutamide’s concentrate was as follows: 0.1 µM during 0–20 passages, 0.5 µM during 21–40 passages, 2.5 µM during 41–60 passages. Cells were observed and photographed with an optical microscope (Mshot, Guangzhou, China).

### Haematoxylin-eosin (HE) staining

After paraformaldehyde prefixed-coomassie, the cells were stained with haematoxylin for 15 min, followed by 1% hydrochloric acid alcohol differentiation for 10 s. Next, samples were held under a tap water flow for 15 min, and stained with eosin for 60 s until a proper stained color and neutral resin was sliced. Cells were observed with an optical microscope (EVOS FL Auto, life technologies, Japan).

### Quantitative real time-polymerase chain reaction (qRT-PCR)

Total RNA was extracted from PCa cell lines by TRIzol reagents from Invitrogen/Life Technologies (Carlsbad, CA, USA) and reverse transcribed to complementary DNA (cDNA) by PrimeScript™ RT reagent Kit with gDNA Eraser (TaKaRa, Dalian, China). The primers were designed by PrimerBank (https://pga.mgh.harvard.edu/primerbank/) and synthesized by GenSys (GenSys, Guangxi, China). AR1, forward, 5’- CCAGGGACCATGTTTTGCC − 3’, reverse, 5’- CGAAGACGACAAGATGGACAA − 3’; AR2, forward, 5’-GACGACCAGATGGCTGTCATT-3’, reverse, 5’- GGGCGAAGTAGAGCATCCT-3’; FKBP5, forward, 5’- CTACACCTGCTGAAGGGACG − 3’, reverse, 5’- CTCCAGCAAACCCTGGTACA − 3’; STEAP1, forward, 5’-ACTGGGCACAATACACGCAT − 3’, reverse, 5’- GGTGACGTCTTCCCAACCAT-3’; ALCAM, forward, 5’-TCCTGCCGTCTGCTCTTCT-3’, reverse, 5’- TTCTGAGGTACGTCAAGTCGG − 3’; ESAM, forward, 5’-GGGGTCACAACAAGCAAACC-3’, reverse, 5’- TTGTCTTGCACATTCACGGAG-3’; ICAM1, forward, 5’- ATGCCCAGACATCTGTGTCC − 3’, reverse, 5’- GGGGTCTCTATGCCCAACAA − 3’; ICAM2, forward, 5’- CGGATGAGAAGGTATTCGAGGT − 3’, reverse, 5’- CACCCACTTCAGGCTGGTTAC − 3’; L1CAM, forward, 5’- TGTCATCACGGAACAGTCTCC − 3’, reverse, 5’- CTGGCAAAGCAGCGGTAGAT − 3’; MCAM, forward, 5’- AGCTCCGCGTCTACAAAGC − 3’, reverse, 5’- CTACACAGGTAGCGACCTCC − 3’; GAPDH, forward, 5’- GGAGCGAGATCCCTCCAAAAT − 3’, reverse, 5’- GGCTGTTGTCATACTTCTCATGG − 3’. qRT-PCR was performed at 95ºC for 600 s, followed by 35 cycles of 95 °C for 15 s, 61 °C for 60 s, and 72 °C for 10 s, melting of 95 °C for 10 s, 65 °C for 60 s, and 97 °C for 1 s, then cooling of 37 °C for 30 s used a LightCvcler 96. Glyceraldehyde-3-phosphate dehydrogenase (GAPDH) was selected as the internal reference. The relative expression of mRNA was calculated by the 2-∆∆Ct method.

### Western blot

Total protein was extracted using cell lysis buffer (Beyotime, catalog number: P00138). Proteins were separated on a 10% SDS‑PAGE gels kit (PG112, Epizyme, Shanghai, China) and transferred onto PVDF membranes (Millipore, Billerica, MA, USA). After blocking with NcmBlot Blocking Buffer (Cat#P30500, NCM Biotech, Suzhou, China), and incubated with the primary antibodies at 4 ˚C overnight, followed by incubation with secondary antibodies (Goat Anti-Rabbit IgG-HRP, Abmart, Shanghai, China). Protein bands were developed with BeyoECL Plus (Beyotime) on an Image Quant LAS500 (GE Healthcare, JAPAN). The primary antibodies against rabbit polyclonal PSA (1:1 000, Abcam ab53774, Cambridge, MA, USA), rabbit monoclonal AR (1:3 000), rabbit monoclonal HK2 (1:3 000), rabbit polyclonal Βaction (1:2 000), (all from Cell Signaling Technology, Beverly, MA, USA). β-Actin was used as the loading control. Enzalutamide (1 μm/lml, A3003, APExBIO, Houston, USA ) was used as an AR inhibitor. The specific bands’ relative densities were quantified using the image detection software Image J software (Version 1.53 h).

### 5-Ethynyl-2’-Deoxyuridine (EdU) assay

The 100 T assay Cell-Light Apollo 567 Stain Kit (Cat#C10371-1, RIBBIO, Guangzhou, China) contains EdU, Apollo component reagent. EdU Assay 100 µl of diluted EdU (1:1000 in RPMI Medium 1640; gibco) was applied in each well of a 96-well plate and incubated in the dark for 4 h. After 100 µl of phosphate-buffered saline (PBS) washing 3 times for 5 min, paraformaldehyde was fixed for 30 min, 100 µl of glycine and 100 µl of Triton X-100 were separated for 10 min and finally, 200 µl of Hoechst was applied and cultured in the dark for 20 min. Eventually, EdU-labeled and Hoechst-stained cells were captured.

### Wound heal assay

The PCa cells were seeded in 6-well plates. Cell growth was allowed to continue until confluence was reached. The cell monolayer was then scratched with a 100 ul micropipette tip and floated cells were washed away with PBS. Cell incubation continued under pour condition medium without FBS to exclude the proliferation influence. And the migration of PCa cells was captured in 48 h. The migration distance and migration area were measured in Image J software (Version 1.53 h).

### Cell counting Kit-8 (CCK-8) assay

At the indicated times, PC3 cells were treated with a CCK8 kit (10 µl/well, NCM, catalog number: C6005) for an additional 1 h. The absorbance was recorded at 450 nm using a microplate absorbance reader EPOCH2 (BIO-TEK, USA).

### Cell adhesion assay

Put the Fibronectin (100 µl/well, Solarbio) or Collagen I (10 µl/well, Solarbio) onto a 96-well microplate for 8 h at 37˚C, and then make the inactivated Fetal Bovine Serum (FBS, 200 µl/well, Solarbio) joined for 1 h at 37 ˚C. PBS buffer was used to wash the well three times and then joined the culture medium to wash again. Then cells were added into the well by an aliquot (1 × 10E4 cells) of the prepared cell suspension for 48 h at 37˚C in a cell culture incubator. After washing none adherent cells, the adherent cells were sustained by a CCK8 kit (10 µl/well) for 4 h and quantified at OD450.

### Cell adhesion signal

The KEGG (https://www.genome.jp/kegg/) database was used to obtain the cell adhesion signaling passway, including 26 genes init: The CCLE (https://portals.broadinstitute.org/ccle/) database was used to find the cell adhesion expression in prostate cancer and prostate tissue cell lines.

### Scanning Electron microscope (SEM)

SEM assay was used to observe the CRPC membrane. The cells were seeded at a density of PC3 and the Lncap-AD cell line was set as a Positive and Negative Control. The CRPC cell lines were extracted from wells, the culture medium was removed and specimens were fixed in 3% glutaraldehyde at 4 ℃. Dehydrated in a series of graded ethanol (50, 70, 80, 90, and 100%) [12]. The specimens were air-dried using a Hitachi HCP-2 critical point dryer (Japan) and sputter-coated with gold using an Eiko IB5 ion coater (Japan). Cell cluster surface morphology was observed with a scanning electron microscope (TESCAN VEGA3, Czech Republic).

### Transmission Electron microscope (TEM)

TEM assay was used to observe CRPC microstructure. The cell samples were collected as an SEM assay. The protocol of digestion and centrifuge of the cells are just like the cell culture. Specimens were fixed in 3% glutaraldehyde and added in 1% osmium oxide buffer. Dehydrated in a series of graded ethanol (30, 50, 70, 80, 90, 95, and 100%). Then, the specimens dealt with the acetone replacement alcohol, epoxy resin permeability, joining the catalyst for polymerization, and ultrathin sectioning. The specimens were observed with a transmission electron microscope (H-7650, HTACHI, Japan), and images were recorded at a magnification of 10 000×.

### Enzyme-linked Immuno sorbent assay (ELISA)

An ELISA kit detected the expression of PSA in the serum. The supernatant was collected by centrifuging tube collected from each group according to the ELISA kit (Human PSA ELISA KIT, mlbio, Shanghai, China). The microplate reader measured the optical density (OD) of each well by Spectrophotometer (EPOCH3, BioTek, USA). The KIT standard sample established the standard curve. The corresponding sample concentration was calculated by standard curve three times.

### Statistics analysis

All data analyses were performed by GraphPad Prism 8 for Windows (version 8.2.1, GraphPad Software, San Diego, California USA, www.graphpad.com). All data were presented as mean ± standard deviation and compared by Student’s t-test, one-way ANOVA, or two-way ANOVA. The Bonferroni test was used for comparing measurement data between groups. *P* < 0.05 was depicted as statistically significant.

## Results

### Morphology of cell culture

**Fig. 1:**
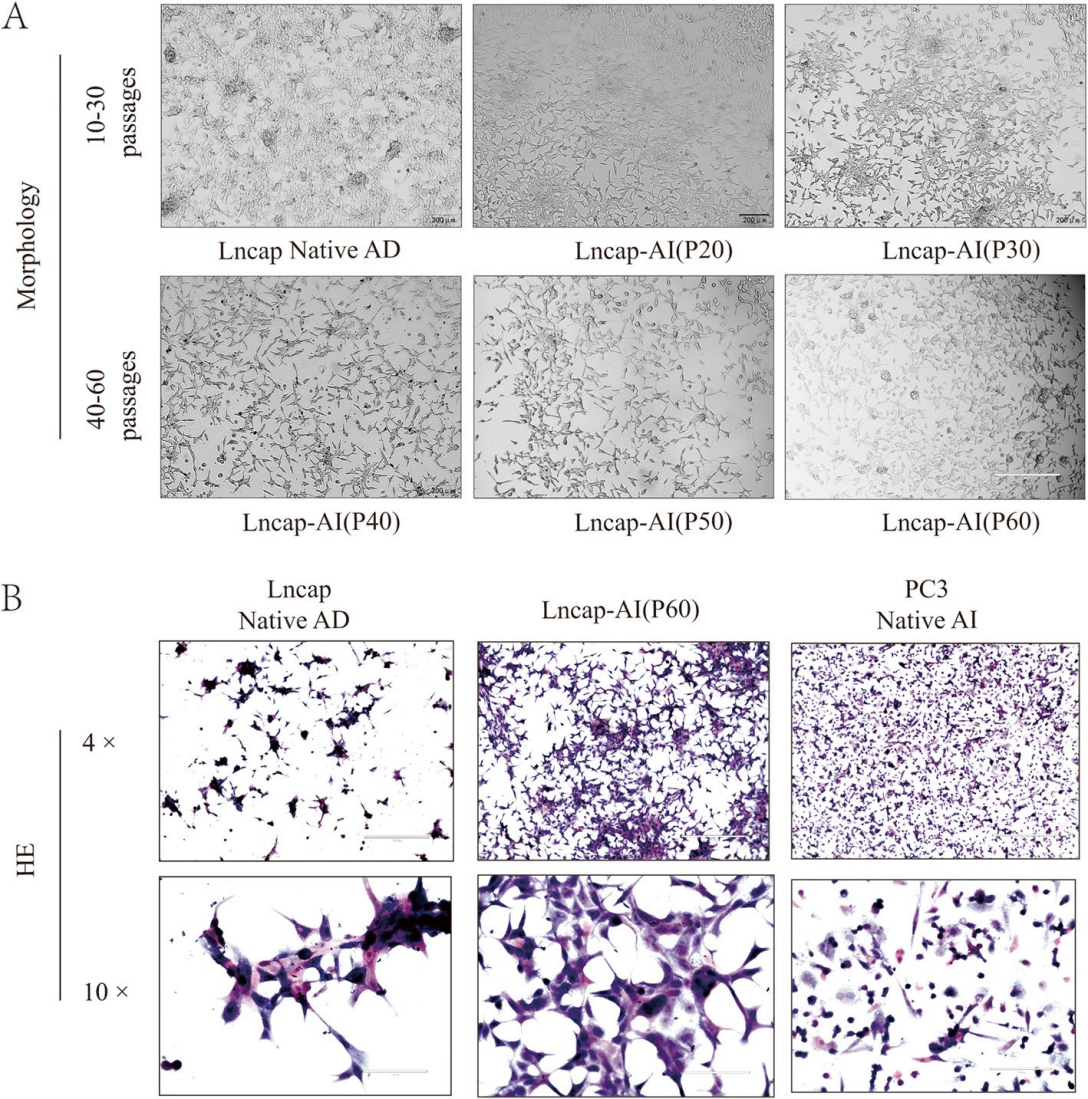
Morphology of CRPC cell culture (**A**) Morphology of Lncap Native AD cells and 20–60 passages of Lncap AI cells. **B** H&E images of CRPC cells

Compared with the Lncap-AD cell line’s morphology, the Lncap-AI cell line changed and each 10 passages morphology was shown in Fig. 1A. In particular passage 60, cell morphology changed a more obvious form slender and left more space between cell-to-cell by androgen deprivation and Flutamide condition. Then we used HE staining to observe the morphology of the Lncap-AI cell line further. The morphology changed from Lncap-AD to Lncap-AI cell line was shown in Fig. 1B. Furthermore, the morphology of the PC3 cell line was stained as a positive androgen-independent cell line. Hence, Lncap-AI cell line’s morphology was different from the Lncap-AD cell line and shown similar to the PC3 cell line in HE staining.

### Identification of Lncap-AI cell line

**Fig. 2:**
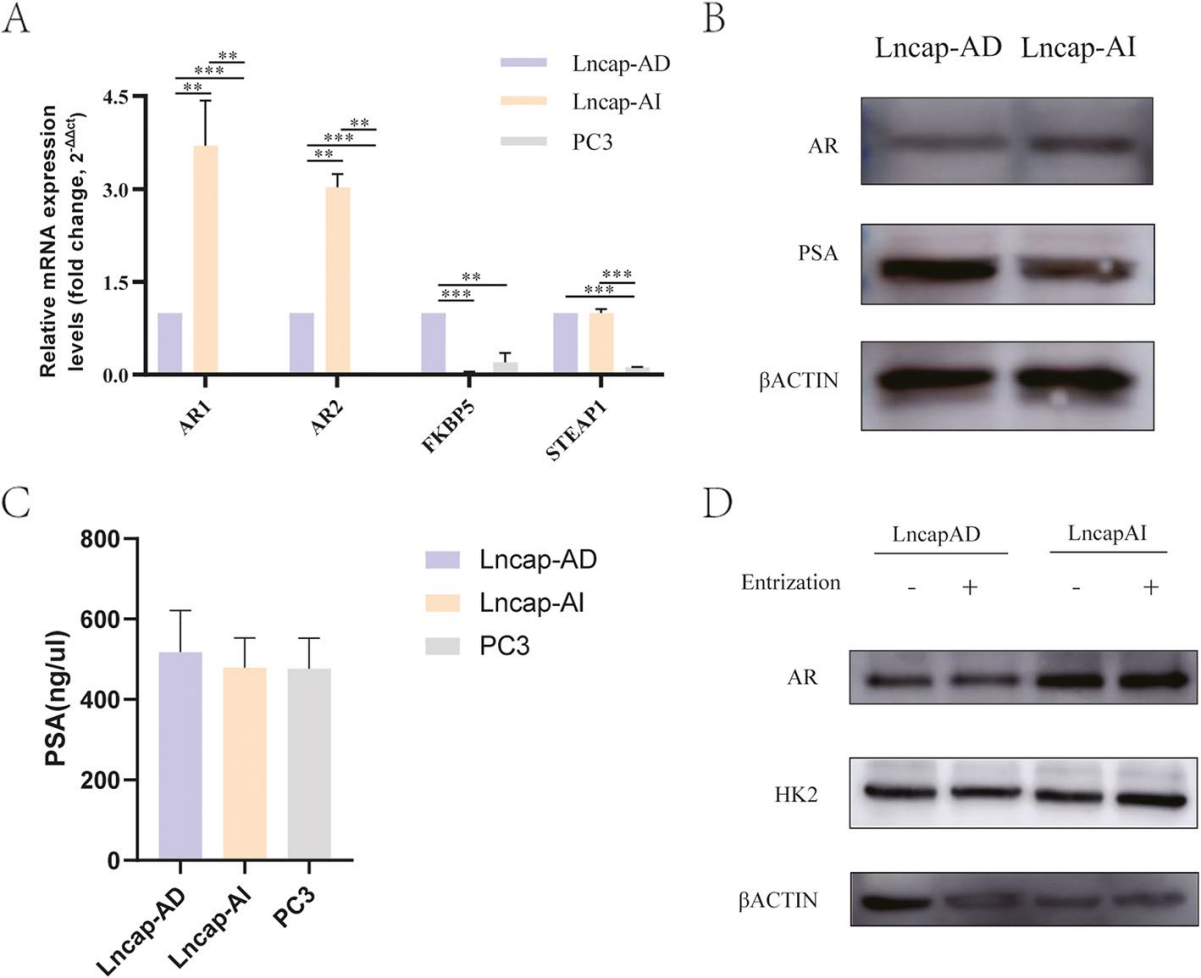
Identification of Lncap-AI cells (**A**) qRT-PCR shows the AR and AR-related genes. **B** The cropping Western Blot shows the PSA and AR protein expression from different parts of the same blot. β-Actin was selected as an internal reference of AR and PSA protein. **C** Serum PSA expression in PCa cells. **D** The cropping Western Blot shows the expression of AR and HK2 by AR inhibitor Enzalutamide. β-Actin was selected as an internal reference for AR and HK2 protein

To identify the formed Lncap-AI cell line, both mRNA and protein levels of AR were detected by qRT-PCR and WB assay. At mRNA level, the expression of AR and related signal pathways (FKBP5 and STEAP1) in the Lncap-AI cell line was significantly higher than in the Lncap-AD cell line (Fig. 2A). The AR protein expression level has shown a lower expression in Lncap-AI cell line than in Lncap-AD cell line (Fig. 2B). Moreover, PSA’s protein expression has been detected to observe the function of three prostate cells and a higher expression in Lncap-AI than Lncap-AD cell line (Fig. 2B). These results suggested that our method was able to form Lncap-AI cell line. PSA secretion is another important detection in the Lncap-AI cell line. At the secretory protein level, we selected PSA to investigate the Lncap-AI cell line secreted ability. In the Elisa test, the Lncap-AI cell line difference showed no significant difference between the Lncap-AD cell line and the PC3 cell line (Fig. 2C). Western blotting analyses were performed in LncapAD and LncapAI cell lines to check the impacts of AR pathway proteins including AR and HK2 were tested, respectively (Fig. 2D). The expression of AR and HK2 proteins were influenced and down-regulation by AR signaling inhibitor Enzalutamide in LncapAD cell line, but no difference in LncapAI cell lines. These results suggested that our method was able to form the Lncap-AI cell line and showed less sensitivity to AR reaction compared to the Lncap-AD cell line.

### Cell function of Lncap-AI cell line

#### Proliferative ability in Lncap-AI cell line

**Fig. 3:**
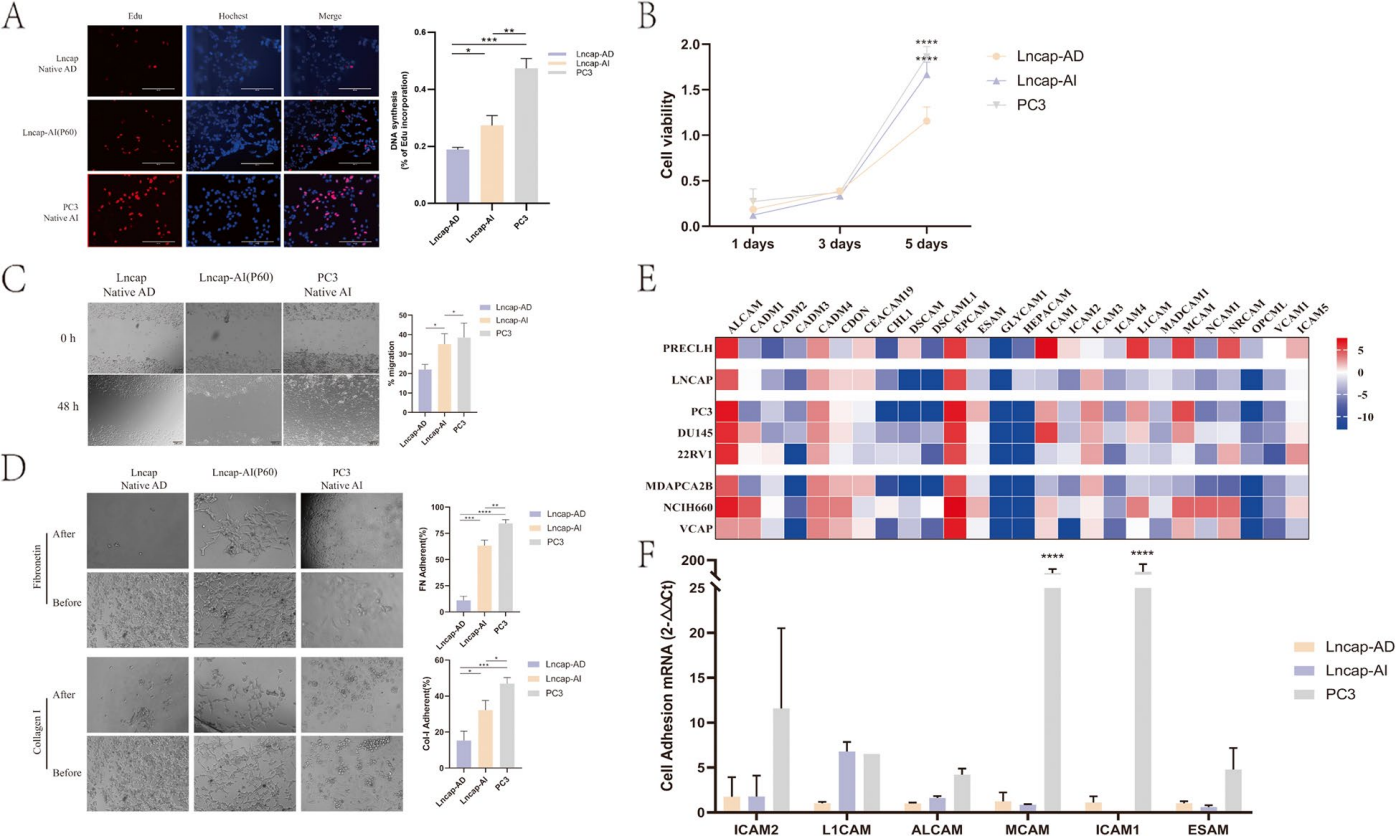
Cell functions detection (**A**) The Edu Assay showed the proliferation of Lncap-AD, Lncap-AI, and PC3 cell lines by Edu(Red), Hochest(Blue), and Merge. **B** Cell viability of three kinds of CRPC cells. Compared to the Lncap-AD cell line. **C** The Wound Healing Assay showed the migration ability of PCa cells. **D** The Adhesion Assay showed the adhesion of Lncap-AD, Lncap-AI, and PC3. **E** The mRNA expression of cell adhesion genes in the CCLE database, including seven prostate cancer cell lines and one prostate tissue cell line. **F** qRT-PCR detected the mRNA expression of three CRPC cell lines. Compared to the Lncap-AD cell line. *p < 0.05, **p < 0.01, ***p < 0.001, ****p < 0.0001

EdU assay was used to observe the proliferation ability further of the Lncap-AI cell line’s biological function. It is suggested that the Lncap-AI cell line improves the proliferation compared to the Lncap-AD cell line (Fig. 3A).

CRPC cells (Lncap-AD, Lncap-AI, and PC3) were sustained by CCK8 kit and analyzed in the same cell concentration at the beginning, and then PC3 cells showed a higher ability to proliferation, Lncap-AI cell line showed a second higher ability and Lncap-AD cell line showed a weak capacity of expansion at 5 days. And compared to the Lncap-AD cell line, the PC3 and Lncap-AI cell lines showed a significant vital difference (Fig. 3B).

#### Migration ability in Lncap-AI cell line

The migration resistances of CRPC cells (Lncap-AD, Lncap-AI, and PC3) were evaluated using a wound-healing assay. It is suggested that the migration of the Lncap-AI cell line showed a significantly farther distance compared to the Lncap-AD cell line (Fig. 3C).

#### Adhesion ability in Lncap-AI cell line

The adhesion assay was used to observe prostate cancer cells’ adhesion ability (Lncap-AD, Lncap-AI, and PC3). The Lncap-AI cell line showed a significantly stable growth ability compared to the Lncap-AD cell line (Fig. 3D). In the CCLE database, 26 genes of cell adhesion signaling were selected from KEGG. They were further detected by the CCLE database (Fig. 3E). 6 genes had a different expression (ICAM2, LICAM, A1CAM, MCAM, LCAM1, ESAM) between Lncap and PC3 were selected to be further detected by qRT-PCR. Compared to the Lncap-AD cell line, MCAM (*p* < 0.001), ICAM1(*p* < 0.001), L1CAM, and A1CAM were overexpressed in mRNA level in Lncap-AI and PC3 cell lines (Fig. 3F).

### Membrane observation of Lncap-AI cell line

**Fig. 4:**
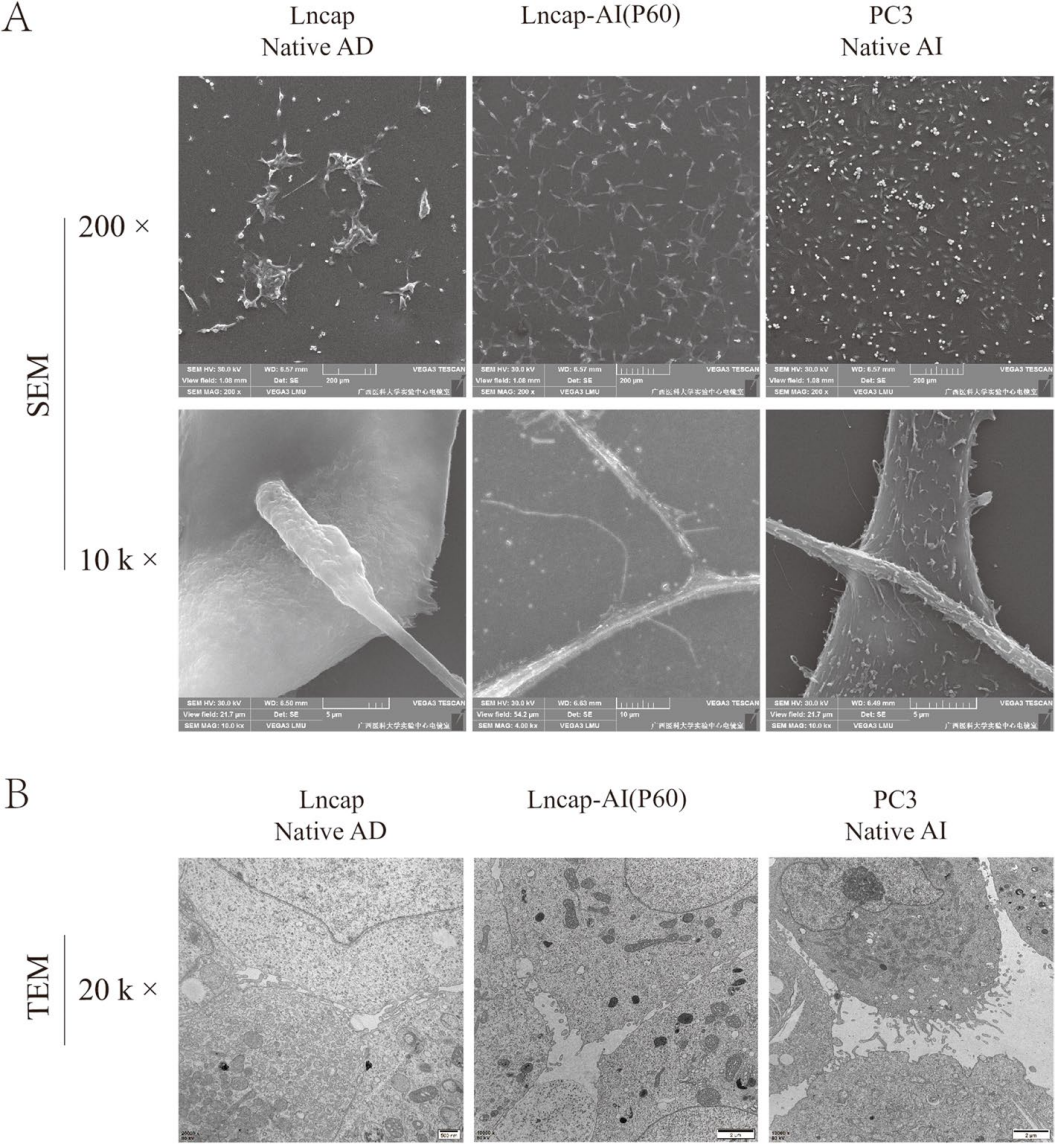
Microstructure of CRPC cells (**A**) SEM images of CRPC cells. SEM: scanning electron microscope. **B** TEM images of CRPC cells. TEM: transmission electron microscope

Scanning Electron Microscope (SEM) technology was used to obtain the cell membrane of CRPC cells. The cell membrane of the Lncap-AI cell line is rougher than that of the Lncap-AD cell line. The surface of the PC3 cell line is not extremely smooth (Fig. 4A). The smoothness of the cell surface may be inversely proportional to the cell adhesion ability. The smoother the cell surface is, the worse the adhesion ability is.

The transmission Electron Microscope (TEM) technology was used to obtain the cell microstructure of CRPC cells. Multiple pseudopodia were observed under TEM micrographs. There are more pseudopods in the Lncap-AI cell line than in the Lncap-AD cell line. The PC3 cell line has a large number of pseudopods (Fig. 4B). A different number of pseudopodia may be the factor leading to the difference in cell adhesion ability.

## Discussion

Castration-resistant prostate cancer (CRPC) is one of the most lethal malignancies worldwide, which owns a poor prognostic and spreads to other organs. Lncap-AI is a potential prostate cancer (PCa) subline model to study CRPC and stimulate the disease’s progression. In the past ten years, the establishment of Lncap-AI is still going on. Lncap cell line transferred to Lncap-AI cell line in androgen-castration environment gradually [4]. Most researchers used the androgen-castration method and a prolonged period of culture to get the Lncap-AI cell line, to adapt to the deprivation androgen environment and could clone proliferation. This protocol has been used in our established process, and some improvements have been made. Flutamide is a nonsteroidal androgen antagonist drug that competes with androgens for androgens receptors. It binds them into a subject complex, which enters the cell’s nuclei and attaches to the nucleoproteins, thereby inhibiting the androgens’ binding to androgens. So androgen deprivation therapy (ADT) and flutamide were used to prevent androgen synthesis and secret from the Lncap-AD cell line.

At the early stage, Lncap-AD cells were spherical, expanded, and gathered into a cluster. After adding the flutamide and culturing with androgen deprivation condition, cell morphology turned slender, leaving more space between cell-to-cell. The details of cell morphology were shown in Fig. 1. HE staining was used to clear morphology and showed the difference between prostate cancer cells (Lncap-AD, Lncap-AI, and PC3). The character of Lncap-AD, a native androgen-dependent cell line, is readily gathered into a cluster and shows a spherical morphology. Unlike Lncap-AD, the Lncap-AI cell line appears a sensible space between cells, and morphology turned to elongated shapes. PC3, a native androgen-independent cell line, showed a separate shape. Both spherical and elongated could be observed. The details of cell morphology were shown in Fig. 1. This modified culture measure could help us obtain a Lncap-AI subline more stable and efficient. Moreover, the expression of AR both in mRNA and protein levels was up-regulation in LncapAI cell lines compared to LncapAD cell lines. As an androgen resistant cell line, LncapAI cell lines showed a poor reaction by AR signaling inhibitor Entrizatimide Fig. 2. These modified cultures and AR expression difference measures could help us obtain a Lncap-AI subline more stable and efficient.

CRPC has been threatened life in the magnificent process, including proliferation [13], apoptosis [14, 15], cell cycle [16], migration and invasion, etc. However, there was less report of the relationship between cell adhesion and other biological behavior, including migration, invasion, EMT, and metastasis. Only researcher Jie Ni et al. [17] focus on the CRPC and cell adhesion ability epithelial cell adhesion molecule (EpCAM) plays an essential role in Prostate Cancer proliferation, invasion, metastasis, and chemo-/radioresistance. The commonly used androgen-independent prostate cancer cell lines include PC3 [18–20] and DU145 [21]. Our research found the Lncap-AI cell line owns more proliferation, migration, and cell adhesion than the Lncap-AD cell line and has the same tendency within CRPC positive cell line-PC3 in Fig. 3.

Moreover, the MCAM (Cell surface glycoprotein MUC18), L1CAM (Neural cell adhesion molecule L1), ICAM1 (Intercellular adhesion molecule 1), and ALCAM (CD166 antigen) were shown the same expression level in CRPC cell lines. MCAM plays an important role in cell adhesion, especially in enhancing hematogenous tumor spread [22]. L1CAM was related to the neural cell adhesion molecule, which could involve cell adhesion and a transmembrane signal at the tyrosine kinase receptor [23]. ICAM1 could promote the endothelial apical cup assembly during the leukocyte trans-endothelial migration period [24]. ALCAM promotes endothelial tube formation by inhibiting endothelial cell migration [25]. All these cell adhesion-related genes show the function between CRPC cell lines. However, the protein levels study needs to be further explored to confirm the cell adhesion function in Fig. 3.

Moreover, some morphology technologies have been used to explore cell adhesion biological behavior, such as a microscope, SEM, TEM, and cell adhesion assay. We investigated cell morphology changes due to exposing prostate cancer cells (Lncap-AD)to androgen deprivation and flutamide. During 10–60 passages, Lncap cells’ morphologywas changed obviously in the sustained culture period. All of the above methods could observe the morphology difference between Lncap-AI and Lncap-AD cell lines obviously in Fig. 4. These findings suggest our established Lncap-AI cell line process has the same tendency the CRPC cell model.

Another important evaluating indicator is PSA secrete ability [26, 27]. In the clinical study, PSA and AR ability were used to detect prostate cancer patients [28]. As we all know, AR is formed in nuclear and expression to the cell cytoplasm [29]. The AR expression is higher in cell nuclear (mRNA level) and the cell cytoplasm (protein level) in the Lncap-AI cell line. Our results were matched to this principle. Moreover, the AR signal pathway-related genes including FKBP5 [30] and STEAP1 [31] were detected, which also showed a strong difference in Lncap-AI and Lncap-AD cell lines. Also, the difference is matched to the PC3 cell line were shown in Fig. 3.

PSA protein was detected to observe malignant prostate cancer in CRPC cell lines. The results showed a higher PSA expression in Lncap-AI than Lncap-AD cell line. These findings suggest our established Lncap-AI cell line process has the ability to secrete PSA. Although our research shows a convenient protocol to prove the Lncap-AI cell line, further studies still need to be used to explore the mechanism of cell adhesion in Fig. 2C.

In conclusion, our research may establish a stable Lncap-AI cell line by flutamide and androgen deprivation conditions. The establishment of cell sublines could simulate the clinical therapy process: prostate cancer from androgen-dependent transferred into androgen-independent condition. And the cell adhesion ability plays an important role in the establishment of transferred function.

## Supplementary Information

**Additional file 1:** **Supplementary Fig. 1.** Blots image.

## Data Availability

All data generated or analyzed during this study are included in this published article.

## Abbreviations

ADT: Androgen deprivation therapy
CCK8: Cell counting kit-8
CRPC: Castration-resistant prostate cancer
EdU assay: 5-ethynyl-2’-deoxyuridine assay
FBS: Foetal fetal bovine serum
GAPDH: Glyceraldehyde-3-phosphate dehydrogenase
Lncap-AD: Lncap androgen-dependent
Lncap-AI: Lncap androgen-independent
OD: Optical density
PCa: Prostate cancer
PSA: Prostate-specific antigen
qRT-PCR: Quantitative real time-polymerase chain reaction
SEM: Scanning electron microscope
TEM: Transmission electron microscope

## References

[1] American Cancer Society (2021). Cancer Statistics 2021 report. J Nucl Med.

[2] Chen M, Ma T, Li J, Zhang HJ, Li Q, Wang JJ, Sang T, Cao CL, Cui XW (2021). Diagnosis of prostate Cancer in patients with prostate-specific Antigen (PSA) in the Gray Area: construction of 2 predictive models. Med Sci Monit.

[3] Siegel RL, Miller KD, Fuchs HE, Jemal A (2021). Cancer Statistics, 2021. CA Cancer J Clin.

[4] Shi XB, Ma AH, Tepper CG, Xia L, Gregg JP, Gandour-Edwards R, Mack PC, Kung HJ (2004). deVere White RW: **molecular alterations associated with LNCaP cell progression to androgen independence**. Prostate.

[5] Buck SAJ, Koolen SLW, Mathijssen RHJ, de Wit R, van Soest RJ (2021). Cross-resistance and drug sequence in prostate cancer. Drug Resist Updat.

[6] Cha S, Shin DH, Seok JR, Myung JK (2017). Differential proteome expression analysis of androgen-dependent and -independent pathways in LNCaP prostate cancer cells. Exp Cell Res.

[7] Abd Wahab NA, Lajis NH, Abas F, Othman I, Naidu R. Mechanism of Anti-Cancer Activity of Curcumin on Androgen-Dependent and Androgen-Independent Prostate Cancer. Nutrients. 2020;12(3):679. 10.3390/nu12030679 PMC7146610 32131560

[8] Cai F, Zhang Y, Li J, Huang S, Gao R. Isorhamnetin inhibited the proliferation and metastasis of androgen-independent prostate cancer cells by targeting the mitochondrion-dependent intrinsic apoptotic and PI3K/Akt/mTOR pathway. Biosci Rep. 2020;40(3):BSR20192826. 10.1042/BSR20192826 PMC7080645 32039440

[9] Voss G, Haflidadottir BS, Jaremo H, Persson M, Catela Ivkovic T, Wikstrom P, Ceder Y (2020). Regulation of cell-cell adhesion in prostate cancer cells by microRNA-96 through upregulation of E-Cadherin and EpCAM. Carcinogenesis.

[10] Yuan M, Yang Y, Li Y, Yan Z, Lin C, Chen J (2020). Mucin-like Domain of Mucosal Addressin Cell Adhesion Molecule-1 facilitates integrin alpha4beta7-Mediated cell adhesion through Electrostatic Repulsion. Front Cell Dev Biol.

[11] Song KH, Kim DM, Lee H, Ham SY, Oh SM, Jeong H, Jung J, Kang CK, Park JY, Kang YM (2021). Dynamics of viral load and anti-SARS-CoV-2 antibodies in patients with positive RT-PCR results after recovery from COVID-19. Korean J Intern Med.

[12] Nanou A, Crespo M, Flohr P, De Bono JS, Terstappen LWMM. Scanning Electron Microscopy of Circulating Tumor Cells and Tumor-Derived Extracellular Vesicles. Cancers (Basel). 2018;10(11):416. 10.3390/cancers10110416 PMC6266016 30384500

[13] Li Q, Lai Y, Wang C, Xu G, He Z, Shang X, Sun Y, Zhang F, Liu L, Huang H (2016). Matrine inhibits the proliferation, invasion and migration of castration-resistant prostate cancer cells through regulation of the NF-kappaB signaling pathway. Oncol Rep.

[14] Evans CP, Lara PN, Jr (2014). Prostate cancer: Predicting response to androgen receptor signalling inhibition. Nat Rev Urol.

[15] Lu K, Liu C, Tao T, Zhang X, Zhang L, Sun C, Wang Y, Chen S, Xu B, Chen M (2015). MicroRNA-19a regulates proliferation and apoptosis of castration-resistant prostate cancer cells by targeting BTG1. FEBS Lett..

[16] Zhang C, Wang L, Wu D, Chen H, Chen Z, Thomas-Ahner JM, Zynger DL, Eeckhoute J, Yu J, Luo J (2011). Definition of a FoxA1 cistrome that is crucial for G1 to S-phase cell-cycle transit in castration-resistant prostate cancer. Cancer Res.

[17] Ni J, Cozzi P, Hao J, Beretov J, Chang L, Duan W, Shigdar S, Delprado W, Graham P, Bucci J (2013). Epithelial cell adhesion molecule (EpCAM) is associated with prostate cancer metastasis and chemo/radioresistance via the PI3K/Akt/mTOR signaling pathway. Int J Biochem Cell Biol.

[18] Cao H, Wang D, Gao R, Chen L, Feng Y (2021). Down regulation of U2AF1 promotes ARV7 splicing and prostate cancer progression. Biochem Biophys Res Commun.

[19] Zhao W, Ning L, Wang L, et al. miR-21 inhibition reverses doxorubicin-resistance and inhibits PC3 human prostate cancer cells proliferation. Andrologia. 2021;53(5):e14016. 10.1111/and.14016 33598946

[20] Garcia-Olivares M, Romero-Cordoba S, Ortiz-Sanchez E, Garcia-Becerra R, Segovia-Mendoza M, Rangel-Escareno C, Halhali A, Larrea F, Barrera D (2021). Regulation of anti-tumorigenic pathways by the combinatory treatment of calcitriol and TGF-beta in PC-3 and DU145 cells. J Steroid Biochem Mol Biol.

[21] Zhang H, Hoang T, Saeed B, Ng SC (1996). Induction of apoptosis in prostatic tumor cell line DU145 by staurosporine, a potent inhibitor of protein kinases. Prostate.

[22] Joshkon A, Heim X, Dubrou C, et al. Role of CD146 (MCAM) in Physiological and Pathological Angiogenesis-Contribution of New Antibodies for Therapy. Biomedicines. 2020;8(12):633. 10.3390/biomedicines8120633 PMC7767164 33352759

[23] Karstens KF, Bellon E, Polonski A, Wolters-Eisfeld G, Melling N, Reeh M, Izbicki JR, Tachezy M (2020). Expression and serum levels of the neural cell adhesion molecule L1-like protein (CHL1) in gastrointestinal stroma tumors (GIST) and its prognostic power. Oncotarget.

[24] Fischer LS, Klingner C, Schlichthaerle T, Strauss MT, Bottcher R, Fassler R, Jungmann R, Grashoff C (2021). Quantitative single-protein imaging reveals molecular complex formation of integrin, talin, and kindlin during cell adhesion. Nat Commun.

[25] Brinkhof B, Zhang B, Cui Z, Ye H, Wang H (2020). ALCAM (CD166) as a gene expression marker for human mesenchymal stromal cell characterisation. Gene X.

[26] Akinboye ES, Brennen WN, Denmeade SR, Isaacs JT (2019). Albumin-linked prostate-specific antigen-activated thapsigargin- and niclosamide-based molecular grenades targeting the microenvironment in metastatic castration-resistant prostate cancer. Asian J Urol.

[27] Wang H, Tai S, Zhang L, Zhou J, Liang C (2019). A calculator based on prostate imaging reporting and data system version 2 (PI-RADS V2) is a promising prostate cancer predictor. Sci Rep.

[28] Al-Khalil S, Boothe D, Durdin T, Sunkara S, Watkins P, Yang S, Haynes A, de Riese W (2016). Interactions between benign prostatic hyperplasia (BPH) and prostate cancer in large prostates: a retrospective data review. Int Urol Nephrol.

[29] Matias PM, Carrondo MA, Coelho R, Thomaz M, Zhao XY, Wegg A, Crusius K, Egner U, Donner P (2002). Structural basis for the glucocorticoid response in a mutant human androgen receptor (AR(ccr)) derived from an androgen-independent prostate cancer. J Med Chem.

[30] Makkonen H, Kauhanen M, Paakinaho V, Jaaskelainen T, Palvimo JJ (2009). Long-range activation of FKBP51 transcription by the androgen receptor via distal intronic enhancers. Nucleic Acids Res.

[31] Alves PM, Faure O, Graff-Dubois S, Cornet S, Bolonakis I, Gross DA, Miconnet I, Chouaib S, Fizazi K, Soria JC (2006). STEAP, a prostate tumor antigen, is a target of human CD8 + T cells. Cancer Immunol Immunother.

