# Supplementary material for: Lncap-AI prostate cancer cell line establishment by Flutamide and androgen-free environment to promote cell adherent
Source: BMC Mol Cell Biol. 2022 Nov 28;23:51. doi: 10.1186/s12860-022-00453-2 (PMC9706963; doi:10.1186/s12860-022-00453-2)
Supplement: Supplementary file 1 — Additional file 1: Supplementary Fig. 1. Blots image. [file 12860_2022_453_MOESM1_ESM.docx]

Supplementary Information

Supplementary Figure 1 blots image.

| AR | 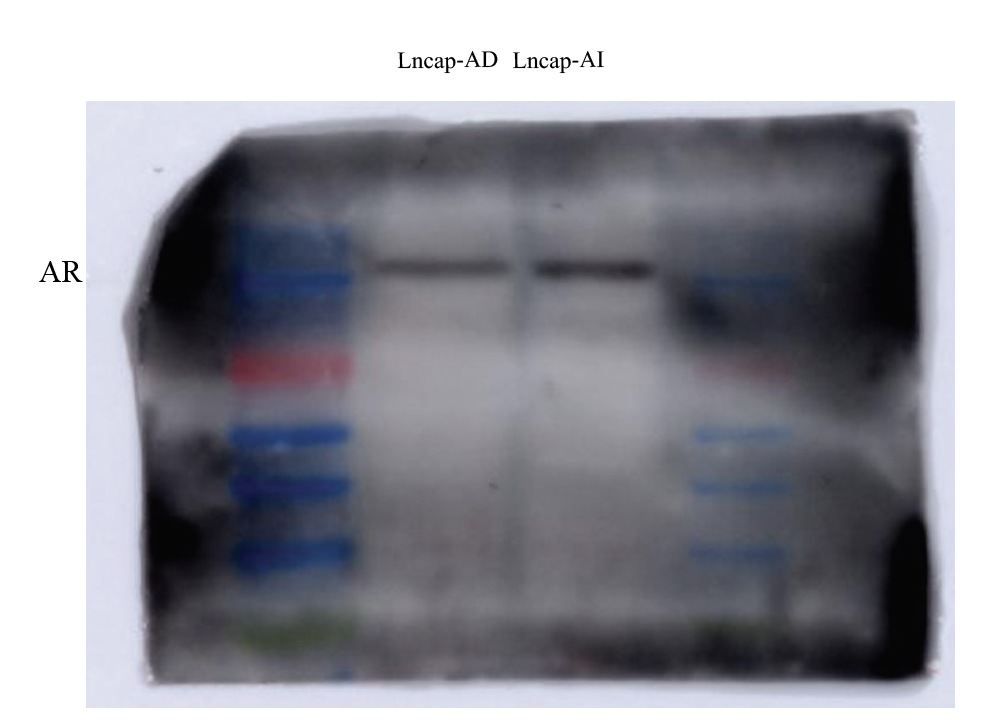 |
| --- | --- |
| PSA | 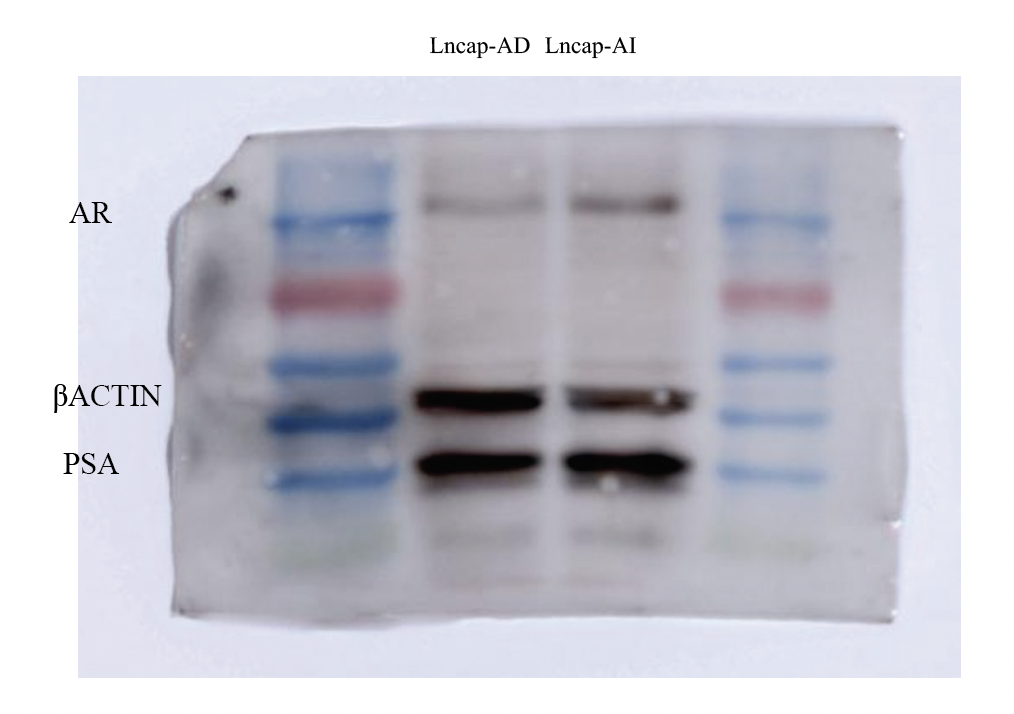 |
| β-Actin | 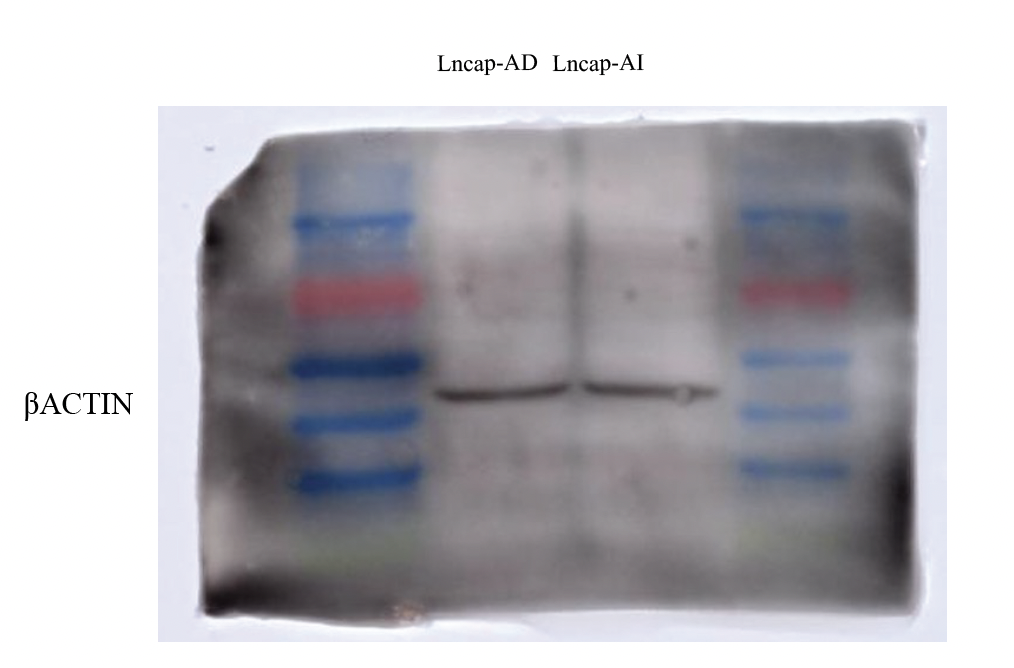 |
| Lncap-AD Lncap-AI AR | 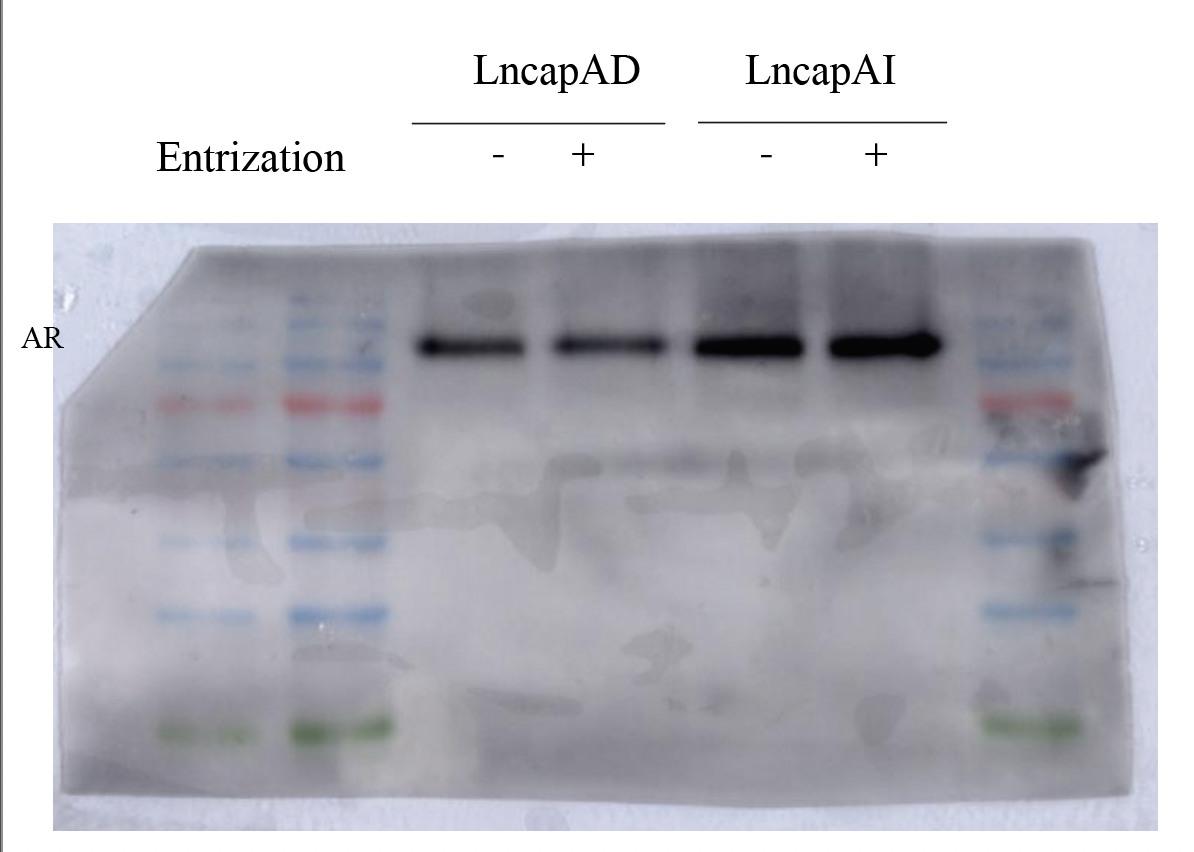 |
| Lncap-AD Lncap-AI HK2 | 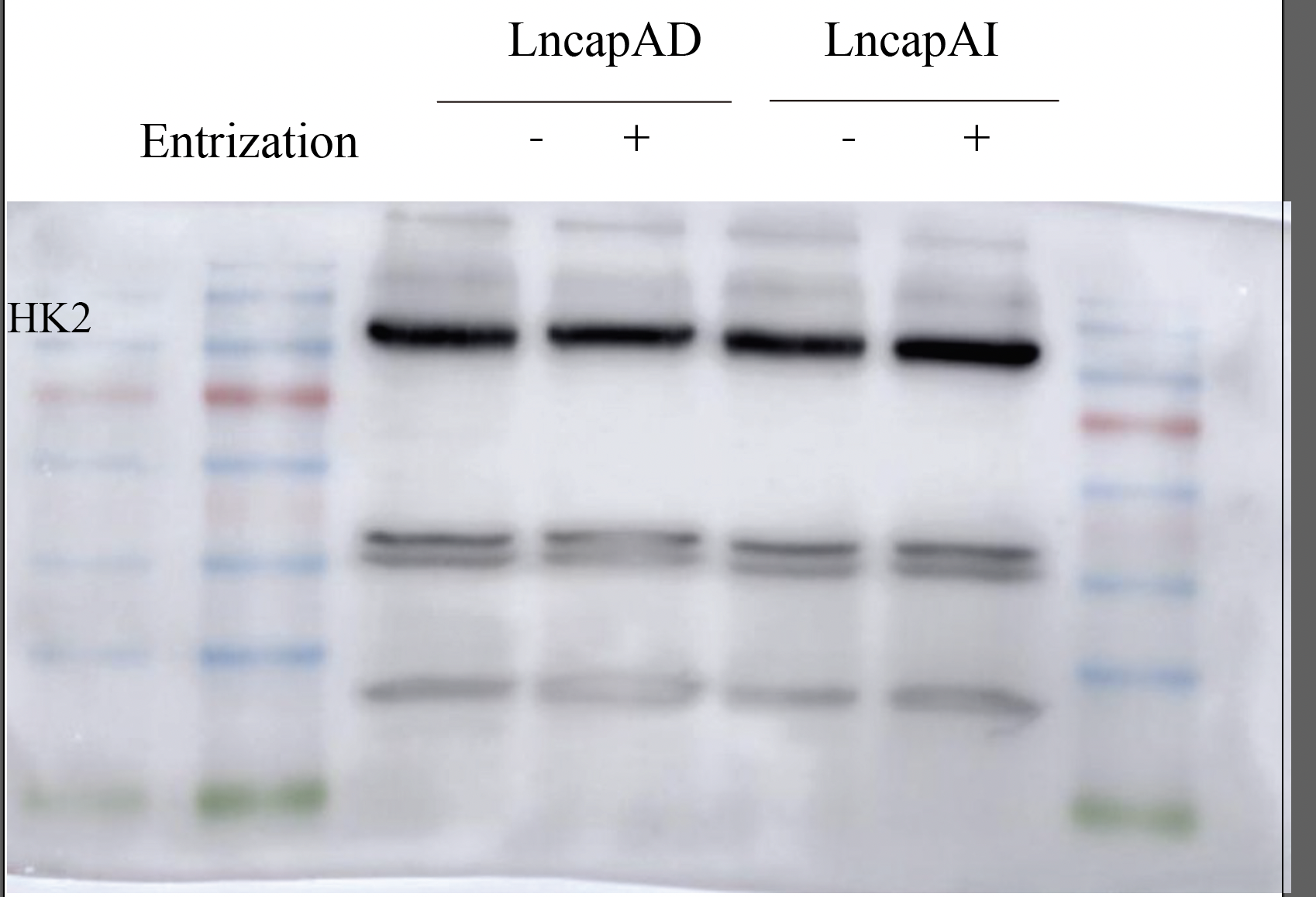 |
| Lncap-AD Lncap-AI  β-Actin | 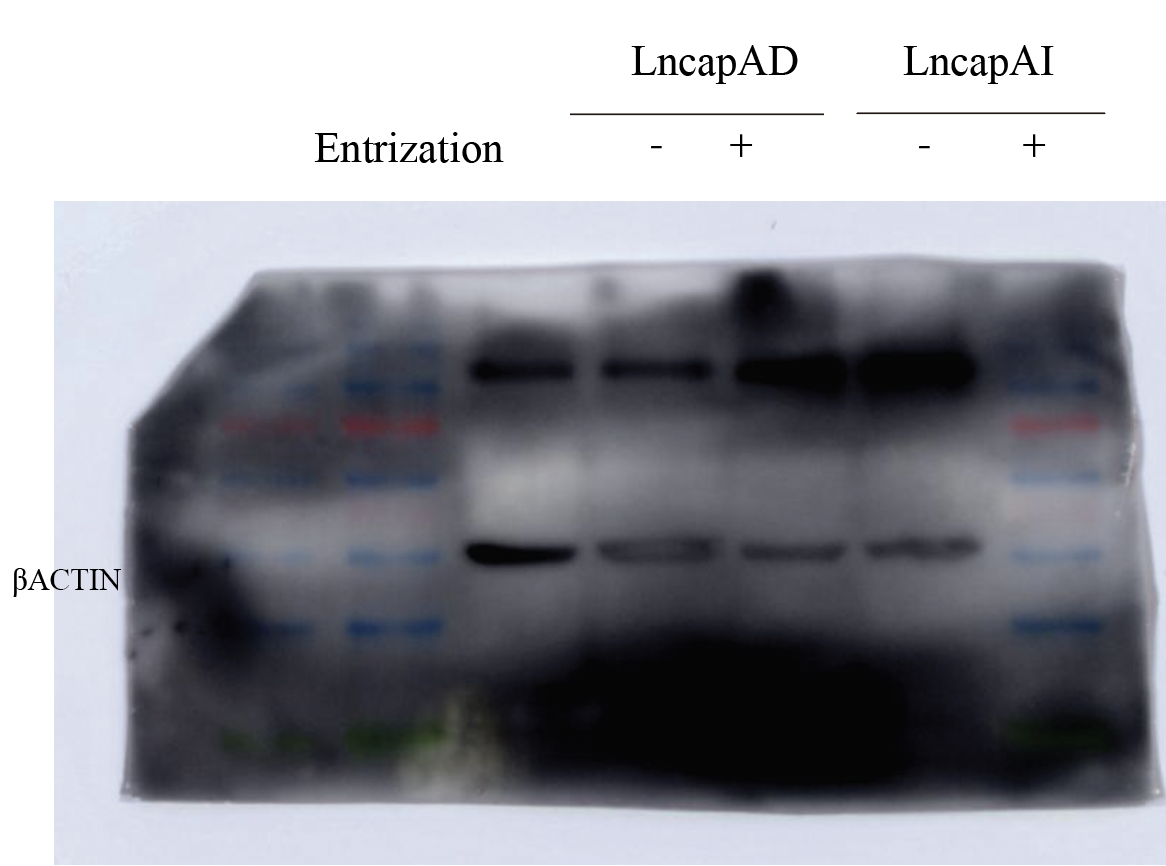 |
